# Clinical, oral immunological and microbiological shifts during and after pregnancy

**DOI:** 10.1007/s00784-023-05408-1

**Published:** 2023-12-29

**Authors:** Pınar Meriç, Angelika Silbereisen, Gülnur Emingil, Veli-Özgen Öztürk, Nagihan Bostanci

**Affiliations:** 1 Department of Periodontology, School of Dentistry, Ege University, İzmir, Turkey; 2 Division of Oral Health and Periodontology, Department of Dental Medicine, Karolinska Institutet, Alfred Nobels Allé 8, Huddinge, 14104 Stockholm, Sweden; 3 Department of Periodontology, School of Dentistry, Adnan Menderes University, Aydın, Turkey

**Keywords:** Cytokines, Oral immunity, Oral microbiota, Periodontal disease, Pregnancy, Saliva

## Abstract

**Objectives:**

Physiological changes and shifts in the oral microbiota composition during pregnancy may affect the maternal immune system. Uncomplicated pregnancy is associated with a T-helper (Th) 2 predominant cytokine regulation (anti-inflammatory), while oral health deterioration during pregnancy is reflected by severe gingival inflammation, a primarily Th1 cytokine phenotype (pro-inflammatory), and oral microbiome alterations. This prospective observational study aimed to evaluate Th cytokine shifts and changes in the oral microbiota composition in saliva of women before and after birth.

**Material and methods:**

Saliva (*n* = 96) was collected before and 6 months after birth, and medical, oral health, and periodontal status were assessed. In a multiplex immunoassay, 10 cytokines were simultaneously analyzed and cumulative Th1 and Th2 cytokine levels and Th1/Th2 ratio were calculated for all groups. Putative periodontal pathogens (*n* = 6) were evaluated by quantitative real-time polymerase chain reaction.

**Results:**

Th2 cytokine levels were significantly lower (*p* = 0.014) while pro-inflammatory cytokine levels were significantly higher (*p* < 0.01) during pregnancy than postpartum. Similar Th1 levels were found between the groups (*p* = 0.143). Th1 and Th2 cytokines positively correlated with periodontal parameters (*p* < 0.001) and levels of studied bacteria during pregnancy (*p* < 0.05).

**Conclusions:**

This study identified a significantly increased Th1/Th2 cytokine ratio during pregnancy and a positive association with putative periodontal pathogens. This immunological and microbiological deregulation in the oral milieu during pregnancy is suggestive of a destructive inflammatory periodontal profile.

**Study registration:**

Clinical Trials.gov (Record BAP-2015).

**Clinical relevance:**

Understanding altered oral immunological and microbiological regulation patterns during pregnancy may help improve the inflammatory periodontal profile in pregnant women.

**Supplementary Information:**

The online version contains supplementary material available at 10.1007/s00784-023-05408-1.

## Introduction

Periodontal diseases are a heterogeneous group of inflammatory diseases of microbial etiology and the most common cause of adult tooth loss. They may contribute to the chronic systemic inflammatory burden with adverse effects for general health, and significant links have been elucidated with diabetes mellitus and higher risk for preterm low-birth weight (PLBW) babies [1]. The associations between adverse pregnancy outcomes and periodontal disease are supported by studies showing that PLBW babies are more common among women with periodontal disease compared to periodontal health [2, 3], though there are also studies demonstrating lack thereof [4–7]. Mechanistically, a possible bi-directional relationship between periodontal disease and pregnancy has been attributed to immune regulators or antioxidants/reactive oxygen species [8–10]. The complex network of cytokines that defines the host immune response against external stimuli consists of pro-inflammatory cytokines and their anti-inflammatory counterparts [11]. However, which of those can act as potential mediators of the association between periodontal diseases and pregnancy warrants further investigation.

T cells are part of the adaptive immune response to external stimuli with T-helper (Th) 1 cells generating a cell-mediated response targeting intracellular pathogens [12], and Th2 cells favoring an antibody-mediated response to target extracellular pathogens [13]. It has been shown that during physiological pregnancy, the Th1/Th2 balance is shifted toward a Th2 bias [14]. A Th1-shifted response however is reportedly associated with recurrent spontaneous abortions [15] and pre-eclampsia [16, 17], though there are studies supporting the opposite (Th2-shifted) response to be associated with these conditions [18, 19]. Th1/Th2 cell balance are associated with recurrent pregnancy losses (RPL), second and third trimester complications [20]. The pregnancy bias toward Th2 system dominance, which protects the fetus, leaves the mother vulnerable to viral infections, which are more effectively contained by the Th1 system [21].

In this study, we aimed to investigate the Th1 and Th2 cytokine profiles in saliva of women during pregnancy and after giving birth (6 months postpartum). The Th1 cytokine set consisted cumulatively of interleukin (IL)-2, interferon-gamma (INF-γ), and tumor necrosis factor-alpha (TNF-α), whereas the set of Th2 cytokines consisted cumulatively of IL-4, IL-5, IL-6, and IL-10. For added value to the analyses, we also investigated the salivary levels of the pro-inflammatory cytokines IL-1β and IL-8, and those of putative periodontal pathogens *P. gingivalis*, *P. intermedia*, *T. denticola*, *T. forsythia*, *F. nucleatum*, and *C. rectus* in saliva.

## Materials and methods

### Study population

A total of 96 pregnant women (aged 19.0 to 40 years) were recruited for this study over a period of 1 year, from the project of Municipality of Bornova, Dental Association and Ege University Medical School Department of Public Health, Izmir, Turkey, as described earlier [22]. General exclusion criteria were any known systemic disease, periodontal treatment within the last 6 months, patients having less than 10 teeth, smokers, and individuals with BMI > 30 kg/m^2^. Additional exclusion criteria in the pregnant group were gestational diabetes mellitus and preeclampsia. Pregnant women were then re-evaluated 6 months postpartum (*n* = 96). The study was conducted in full accordance with the ethical principles of the World Medical Association Declaration of Helsinki. The study was approved by the Ethics Committee of Ege University (protocol number 13–3.3/8) and conforms to STROBE guidelines for observational studies [23]. The study protocol was explained to the participants, and written informed consent was obtained from each one of them prior to registering medical and dental histories, clinical periodontal examination, and saliva sampling. The study design included the following groups: pregnant women (*n* = 96) who were in their second trimester (weeks 16–24) or third trimester (weeks 25–34), postpartum women (*n* = 96) who were evaluated 6 months after giving birth (Supplementary Fig. 1). Further analyses were performed by grouping the participants based on infants with low birth weight (*n* = 7) and infants with normal birth weight (*n* = 89).

### Collection and processing of saliva samples

Whole unstimulated saliva samples were obtained simply by expectorating into polypropylene tubes prior to clinical periodontal measurements or any periodontal intervention. This was performed during morning sessions, following overnight fasting during which subjects were requested not to drink (except water) or chew gum. The individuals were asked to rinse their mouth with tap water before expectorating whole saliva into sterile 50-ml tubes for 5 min. The saliva samples were then placed on ice, supplemented with EDTA-free Protease Inhibitor Cocktail (Roche Applied Science, Switzerland) prior to centrifuging at 10,000 × *g* for 15 min at 4 °C. The resulting supernatants were immediately aliquoted and frozen (− 80 °C) until the analysis.

### Clinical periodontal measurements

After saliva sampling, clinical periodontal recordings, including full mouth dichotomous ( ±) plaque index (FMPI, in %), probing pocket depth (FMPD, in mm), and dichotomous ( ±) presence of bleeding on probing (FMBOP, in %, occurring within 15 s after periodontal probing) were performed at 6 sites on each tooth present (except third molars) using a Williams periodontal probe (Hu Friedy, Chicago, IL, USA). Clinical attachment level (CAL) was assessed from the cement-enamel junction to the base of the probable pocket. All clinical measurements were performed by a single calibrated examiner (P.M.).

### Multiplex immunoassay and enzyme-linked immunosorbent assay

A commercially available Cytokine Human Ultrasensitive Magnetic 10-Plex Panel (Novex®, ThermoFisher Scientific, USA) multiplex immunoassay was used to measure cytokine levels in salivary supernatants on the Luminex®200 platform as described earlier [24]. This assay was able to simultaneously measure granulocyte–macrophage colony-stimulating factor (GM-CSF), IFN-γ, IL-1β, IL-2, IL-4, IL-5, IL-6, IL-8, IL-10, and TNF-α with minimum sensitivities of 0.01, 0.01, 0.05, 0.05, 0.1, 0.1, 0.5, 0.05, 0.05, and 0.5 pg/ml, respectively, according to the manufacturer. The set of Th1 cytokines consisted cumulatively of IL-2, INF-γ, and TNF-α, whereas the Th2 cytokine set consisted cumulatively of IL-4, IL-5, IL-6, and IL-10. In addition, levels of individual pro-inflammatory cytokines IL-1β and IL-8 were measured in saliva by commercially available enzyme-linked immunosorbent assays (ELISA) (R&D systems Duo-set ELISA kits, R&D Systems, Inc., Minneapolis, USA).

### Microbiological analyses

Salivary periodontal pathogens such as *F. nucleatum*, *P. gingivalis*, *P. intermedia*, *T. denticola*, *C. rectus*, and *T. forsythia* were detected by quantitative real-time polymerase chain reaction (qPCR) according to protocols established earlier [25]. Bacterial DNA of known amounts was extracted from laboratory grown cultures of the selected species to generate standard curves to estimate the number of copies of each species in unknown saliva samples. The theoretical bacterial numbers in each sample were calculated based on the measured DNA amount and the estimated genome weight as described earlier [26].

### Statistical analysis

A power analysis was conducted for this study identifying that a minimum cohort of 67 patients was deemed necessary for each group to attain 80% statistical power, maintaining a significance level of 0.05 to mitigate the risk of a type 1 error, while accommodating a medium effect size of *d* = 0.5. The distribution of the data was validated by D’Agostino-Pearson omnibus normality test and statistical analysis was performed using non-parametric methods. Comparisons between all groups were made using the Wilcoxon test. Statistical analyses were conducted using the statistical software (GraphPad Prism version 6.00c for Mac OS X, GraphPad Software, La Jolla California USA), and statistical significance was considered at *p* < 0.05. Correlations between parameters were analyzed by Spearman’s correlation test.

## Results

### Clinical findings

Clinical periodontal measurements of the subjects among all study groups are outlined in Table 1. The data indicate no significant differences in periodontal disease status during pregnancy (baseline) and postpartum. The prevalence of periodontal diseases was 83% among pregnant women (71 gingivitis, 9 periodontitis, 16 periodontal health). None had experienced preterm birth or spontaneous abortion, whereas 7 out of 96 (7.29%) infants showed low birth weight (< 2500 g). The clinical periodontal parameters of mothers with low birth weight infants compared to those with normal birth weight did not differ (*p* > 0.05) (Table 1).

**Table 1:** Clinical periodontal measurements in the study groups

	Pregnant *N* = 96	Postpartum *N* = 96
FMPD (mm)	2.0 (1.0)	2.0 (1.0)
FMBOP (%)	50.0 (42.5)	50.0 (40.0)
FMPI (%)	50.0 (50.0)	50.0 (40.0)
Baby weight	–	3655 (636.25)
	Low birth weight *N* = 7	Normal birth weight *N* = 89
FMPD (mm)	1.0 (1.0)	2.0 (1.0)
FMBOP (%)	40.0 (50.5)	50.0 (50.0)
FMPI (%)	40.0 (50.0)	50.0 (50.0)

Median and IQR are given

### Biochemical findings

**Fig. 1:**
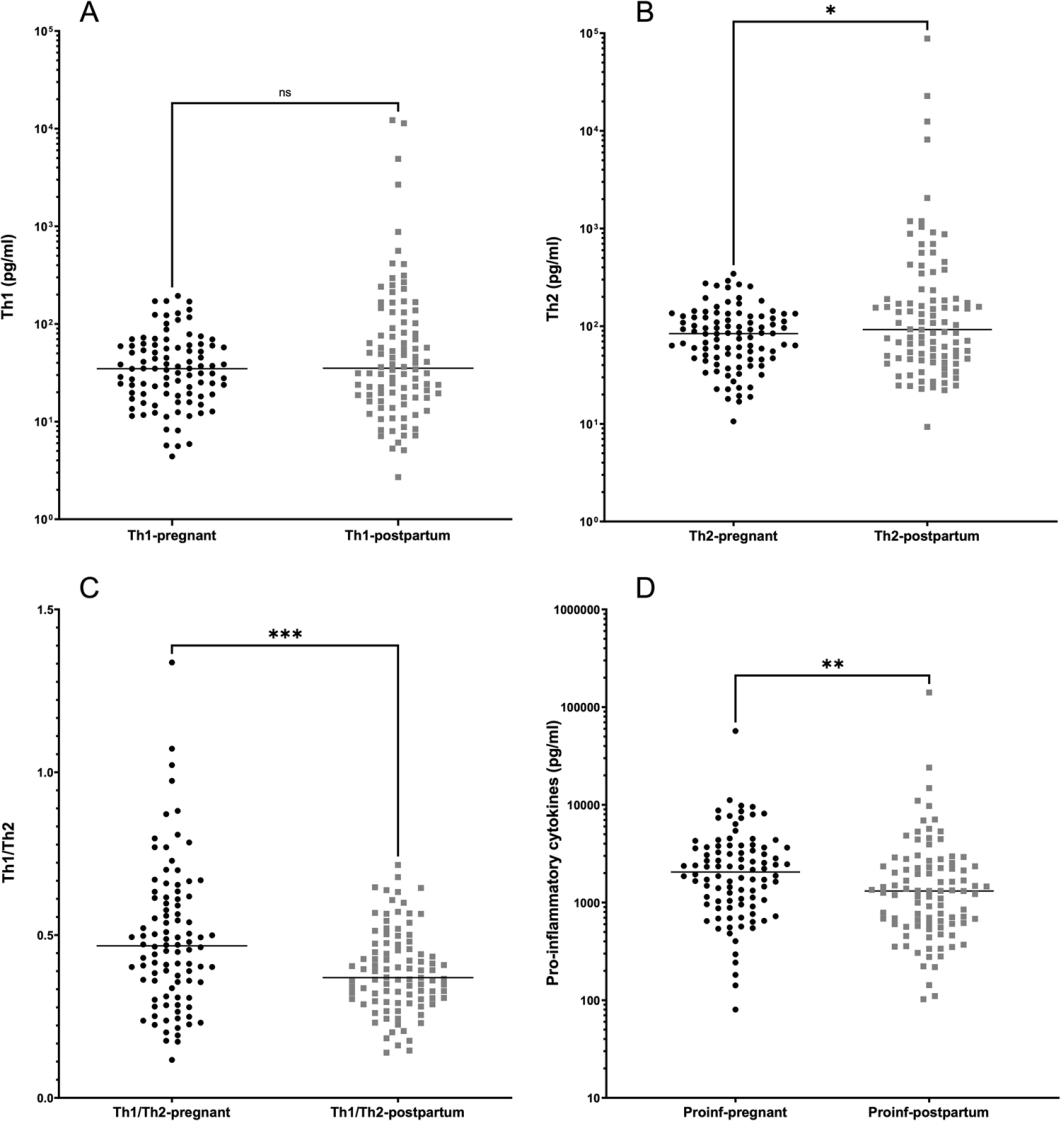
Salivary biochemical findings in the pregnant (*n* = 96) and postpartum (*n* = 96) groups. **A** Th1 cytokine levels (IFN-γ, IL-2, TNF-α) (*p* = *0.14*); **B** Th2 cytokine levels (IL-4, IL-5, IL-6, IL-10) (*p* = *0.015*); **C** Th1/Th2 ratio (*p* = *0.0004*); **D** proinflammatory cytokines (IL-1β, IL-8 levels) (*p* = *0.01*). Median and IQR are given. *p < 0.05, **p < 0.001

Salivary levels of IL-1β, TNF-α, IL-4, and IL-6 could be detected in all samples. Samples below the detection limit for all other cytokines were assigned the value 0. Salivary analysis demonstrated that Th1 (IL-2, INF-γ, and TNF-α) levels were similar between the pregnant and the postpartum groups (*p* = 0.143, Fig. 1A), whereas Th2 (IL-4, IL-5, IL-6, and IL-10) levels were significantly higher after pregnancy (*p* = 0.014, Fig. 1B). As a result, the relative Th1/Th2 ratio was significantly higher during pregnancy (*p* < 0.001, Fig. 1C). When individual pro-inflammatory cytokines (IL-1β and IL-8) (Fig. 1D) were investigated, they were significantly higher during than after pregnancy (*p* < 0.01). Details about the salivary levels of each detectable cytokine in the pregnant and postpartum groups are presented in the Supplementary Table 2. Comparing biochemical data between low birth weight and normal birth weight groups did not reveal any significant differences (Supplementary Table 3). Details about Th1, Th2, proinflammatory cytokines, and Th1/Th2 ratio levels according to the periodontal status in pregnant and postpartum groups are given in the Supplementary Table 4.

In addition, associations between biochemical and clinical periodontal parameters were investigated in the different study groups. In the pregnant group (Table 2), Th1, Th2, and pro-inflammatory cytokine levels (IL-1β and IL-8) were positively correlated with all periodontal parameters (FMPD, FMBOP, and FMPI) (Th1: *r* = 0.363, *r* = 0.455, *r* = 0.443, respectively (all *p* < 0.0001); Th2: *r* = 0.331, *r* = 0.425, *r* = 0.426, respectively (all *p* < 0.0001); pro-inflammatory: *r* = 0.247, *r* = 0.364, *r* = 0.358, respectively (all *p* < 0.05)). In the postpartum group (Table 2), no significant correlations between biochemical and clinical periodontal parameters were observed (*p* > 0.05).

**Table 2:** Correlation analysis between clinical periodontal parameters and salivary biomarkers in the pregnant and postpartum groups

Pregnant	Correlation	FMPD (mm)	FMBOP (%)	FMPI (%)
Th1	r	0.363	0.455	0.443
	p	< 0.0001*	< 0.0001*	< 0.0001*
Th2	r	0.331	0.425	0.426
	p	< 0.0001*	< 0.0001*	< 0.0001*
Th1/Th2 ratio	r	0.164	0.198	0.172
	p	0.109	0.053	0.094
Pro-inflammatory cytokines	r	0.247	0.364	0.358
	p	0.015*	< 0.0001*	< 0.0001*
Postpartum	Correlation	FMPD (mm)	FMBOP (%)	FMPI (%)
Th1	r	0.363	0.326	0.111
	p	0.282	0.403	0.343
Th2	r	0.122	0.145	0.143
	p	0.238	0.158	0.164
Th1/Th2 ratio	r	-0.006	-0.142	-0.042
	p	0.955	0.647	0.684
Pro-inflammatory cytokines	r	-0.018	-0.061	-0.046
	p	0.864	0.552	0.655

*FMPD* full mouth probing depth, *FMPI* full mouth plaque index, *FMBOP* full mouth bleeding on probing; Th1: IFN-γ, IL-2, TNF-α; Th2: IL-4, IL-5, IL-6, IL-10; pro-inflammatory cytokines: IL-1β, IL-8

*Bold indicates statistically significant *p*-values

### Microbiological findings

**Fig. 2:**
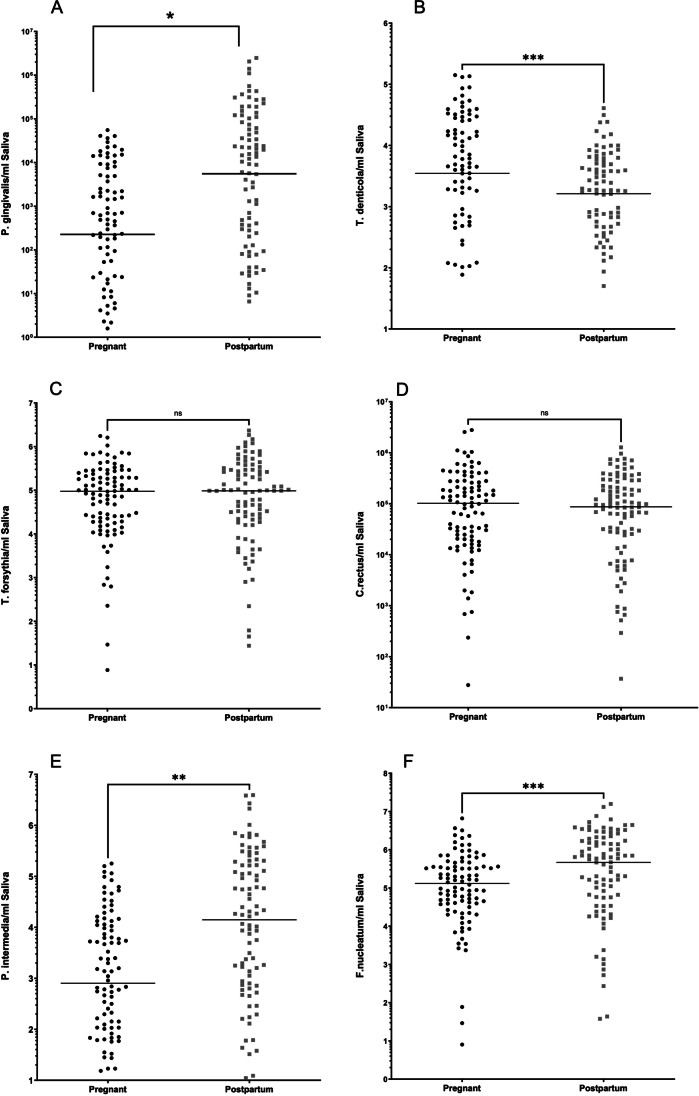
Microbiological findings in the pregnant (*n* = 96) and postpartum (*n* = 96) groups. **A** *P. gingivalis* (*p* = *0.0001*), **B** *T. denticola* (*p* = *0.0001*), **C** *T. forsythia* (*p* = *0.30*), **D** *C. rectus* (*p* = *0.28*), **E** *P. intermedia* (*p* = *0.0012*), and **F** *F. nucleatum* (*p* = *0.0005*). Median and IQR are given. *p < 0.05

The salivary levels of selected putative periodontal pathogens were also investigated during pregnancy and postpartum. Salivary levels of *P. gingivalis* (Fig. 2A), *P. intermedia* (Fig. 2E), and *F. nucleatum* (Fig. 2F) were significantly higher postpartum than during pregnancy (*p* < 0.001), whereas the opposite was the case for *T. denticola* (*p* = 0.004, Fig. 2B). No significant differences in the levels of *T. forsythia* (Fig. 2C) and *C. rectus* (Fig. 2D) between the pregnancy and postpartum groups were observed (*p* = 0.363 and *p* = 0.515, respectively). Comparing microbiological data between low birth weight and normal birth weight groups did not reveal any differences (*p* > 0.05, Supplementary Table 3).

In addition, associations between biochemical parameters and salivary microbiota were investigated in the different study groups. A positive correlation between either Th1 or Th2 levels and all studied bacterial species (*P. gingivalis*, *P. intermedia*, *P. denticola*, *T. forsythia*, *F. nucleatum*, *C. rectus*) was revealed during pregnancy (Th1: *r* = 0.251, *r* = 0.280, *r* = 0.365, *r* = 0.401, *r* = 0.379, *r* = 0.408, respectively (all p < 0.05); Th2: *r* = 0.200, *r* = 0.229, *r* = 0.370, *r* = 0.369, *r* = 0.295, *r* = 0.377, respectively (all *p* < 0.05)) (Table 2). In the postpartum group, both Th1 and Th2 levels positively correlated only with *T. forsythia* and *C. rectus* levels (Th1: *r* = 0.265 and *r* = 0.306, respectively (both *p* < 0.05); Th2: *r* = 0.240 and 0.233, respectively (both *p* < 0.05) (Table 3).

**Table 3:** Correlation analysis between biochemical parameters and salivary microbiota in the pregnant and postpartum groups

Pregnant	Correlation	Th1	Th2
*P. gingivalis*	r	0.251	0.200
	p	0.014*	0.049*
*P. intermedia*	r	0.280	0.229
	p	0.006*	0.025*
*T. denticola*	r	0.365	0.370
	p	< 0.0001*	< 0.0001*
*T. forsythia*	r	0.401	0.369
	p	< 0.0001*	< 0.0001*
*F. nucleatum*	r	0.379	0.295
	p	< 0.0001*	< 0.0001*
*C. rectus*	r	0.408	0.377
	p	< 0.0001*	< 0.0001*
Postpartum	Correlation	Th1	Th2
*T. forsythia*	r	0.265	0.240
	p	0.009*	0.019*
*C. rectus*	r	0.306	0.233
	p	0.002*	0.022*

Th1: IFN-γ, IL-2, TNF-α; Th2: IL-4, IL-5, IL-6, IL-10

*Bold indicates statistically significant *p*-values.

## Discussion

In the current work, we have studied the levels of pro- and anti-inflammatory cytokines and various oral microbes in saliva during and after pregnancy. Our results showed a bias toward higher Th2 cytokine levels in saliva during pregnancy, but no significant alterations in Th1 cytokines. While various studies support the requirement of a Th1 to Th2 response shift for a successful pregnancy outcome, others do not [27]. In 1993, Wegmann et al. created the “Th2 phenomenon” where the Th1/Th2 activity balance is strongly shifted toward a Th2 response during pregnancy [14]. According to that hypothesis, a Th1 response is incompatible with a successful pregnancy outcome [28]. Predominant Th1-type immunity has been observed in recurrent spontaneous abortion [28–30] and, more precisely, circulating levels of TNF-α and IFN-γ (Th1 cytokines) were reported to be higher in patients with a subsequent miscarriage than in women with a successful pregnancy [31–33]. However, a recent study suggested that the Th1/Th2 balance in pregnancy is shifted toward a Th1 suppression rather than a Th2 promotion [34]. Meaning a successful pregnancy outcome may therefore require a predominant Th2-type cytokine response, while a poor pregnancy outcome may be associated with an increase in Th1 cytokines [35]. Nevertheless, both overstimulation of Th1 or Th2 immunity could be rather harmful, and a regulated cytokine network and Th1/Th2 cooperation might be crucial for the maintenance and successful outcome of a pregnancy [36, 37].

According to our results, the Th1/Th2 cytokine ratio was significantly higher during pregnancy than postpartum with a significant shift from a Th1/Th2 balance to a Th2 bias in the pregnant group. Thus, the Th1/Th2 ratio works as a marker for a successful or failing pregnancy, but one must keep in mind other factors involved in this complex mechanism during pregnancy establishment and maintenance [38]. In this line, studies supported a possible bi-directional relationship between oral health and pregnancy [8, 9], with poor oral health being considered an important risk factor for a poor pregnancy outcome [39]. During pregnancy, the occurring physiological changes and shifts in the composition of oral microbiota are affecting the maternal immune system [8, 40]. This may be reflected in the levels of disease markers in saliva with or without a respective clinical presentation. It was found that levels of IL-1β and prostaglandin E2 in gingival crevicular fluid (GCF) were not affected during pregnancy, but both markers were higher in pregnant than in non-pregnant women [41]. However, this could not be explained with exacerbated gingival inflammation during pregnancy. Furthermore, gene expression analyses of various Th1 and Th2 cytokines in GCF (IL-1α, IL-1β, IL-8, and TNF-α) and in gingival tissue (IL-1β, IL-6, and TNF-α) during pregnancy and compared to postpartum with and without periodontal disease were not affected and could not be related to periodontal parameters such as bleeding scores [42, 43]. While our results are in line with these previous findings in postpartum women showing no association between salivary cytokine levels and periodontal parameters, on the contrary, our results revealed that during pregnancy, Th1, Th2, and pro-inflammatory cytokine levels in saliva were positively correlated with plaque index, bleeding index, and probing depths. Earlier studies reported that clinical periodontal parameters might worsen during pregnancy, even without a concomitant increase in plaque index, which decreases after delivery [44]. Our findings did not show significant differences in any of the investigated periodontal parameters between pregnancy and postpartum. Another possible explanation could be a shifting oral microbial composition during pregnancy [40]. Furthermore, while we did not investigate the levels of the markers under consideration as gestation advanced, it is important to acknowledge that there can be alterations in saliva protein levels during pregnancy. As elucidated by [45], the observed elevation in salivary estrogen levels during the second and third trimesters theoretically suggests a potential impact on the expression profiles of specific microbial and protein markers. In line with this, nitric oxide increased with advancing gestation, but the oxidative stress and the antioxidant capacity did not change significantly during pregnancy [46]. Some of the antimicrobial peptides displayed dynamic fluctuations, while others maintain a consistent pattern throughout the course of pregnancy and postpartum assessments [47].

To further investigate the potential of microbial shifts in the oral flora before and after delivery, we have quantified bacterial saliva levels of *P. gingivalis*, *P. intermedia*, *T. denticola*, *T. forsythia*, *F. nucleatum*, and C*. rectus*. According to our findings, salivary levels of *T. denticola* were significantly higher during pregnancy while *F. nucleatum*, *P. gingivalis*, and *P. intermedia* were significantly higher in postpartum women. Furthermore, salivary Th1 and Th2 cytokine levels were positively correlated with all five bacterial species in pregnant women, and with *T. forsythia and C. rectus* levels postpartum. The physiological changes associated with pregnancy can lead to shifts in the microbial communities colonizing mucosa surfaces, and bacteria and their products can elicit an intra-amniotic inflammatory response and subsequently induce the production of pro-inflammatory cytokines and chemokines [8, 48]. Pregnant women in different gestational phases demonstrated an increase in Proteobacteria and Actinobacteria abundance from the first to the trimester and a decrease in microbial richness, which also persisted for 1 month postpartum [48]. Furthermore, increased levels of sex-steroid hormones in pregnant women were associated with dental biofilm including species of Fusobacterium and Prevotella, *Streptococcus anginosus* and *Streptococcus intermedius* [49]. The proportions of certain putative periodontopathogens such as *Aggregatibacter actinomycetemcomitans* (*A. actinomycetemcomitans*) and *Parvimonas micra* were also shown to be increased during pregnancy [40]. However, since it is known that levels of sex-steroid hormones in pregnant women can reflect the magnitude of gingival inflammation, this study would have profited from investigations into salivary steroid hormone levels in saliva of those pregnant women [45, 49].

In a case–control study, levels of oral bacteria species (including the red complex and *A. actinomycetemcomitans*, *S. mutans*) tended to increase during pregnancy especially in mothers with preterm birth, while these levels remained stable in mothers with a term delivery [50, 51]. Our findings however did not identify any significant differences in Th1, Th2, and pro-inflammatory cytokine levels or periodontal pathogen levels between low and normal birth weight infants. Nevertheless, all women with low birth weight infants presented with gingivitis. As several risk factors can influence birth weight including poor oral health, each of them may have a rather small individual impact or the sample size may not be sufficient to detect significant effects [52]. Despite that the strength of the association found between periodontal diseases and birth weight varies between studies, it is agreed that maintaining oral health during pregnancy is beneficial to the mother and her infant.

## Conclusions

This study identified a significant salivary shift in the Th1/Th2 balance between pregnancy and postpartum, which is driven by an increase in Th2 cytokine levels during postpartum. Furthermore, Th1 and Th2 cytokine levels are positively correlated with clinical periodontal parameters and specific oral microbiota in saliva of pregnant women. Understanding the altered immunological and microbiological regulation patterns in the oral milieu during pregnancy may help to better control and improve the inflammatory periodontal profile in pregnant women.

### Supplementary Information

Below is the link to the electronic supplementary material.

Supplementary file1 (DOCX 97 KB)

## Data Availability

All data generated and analyzed during this study are included in this published article and the supplementary material.

## Declarations

### Disclaimer

The funders had no role in study design, data collection and analysis, decision to publish, or preparation of the manuscript.
