# Supplementary material for: Clinical, oral immunological and microbiological shifts during and after pregnancy
Source: Clin Oral Investig. 2023 Dec 29;28(1):60. doi: 10.1007/s00784-023-05408-1 (PMC10756889; doi:10.1007/s00784-023-05408-1)
Supplement: Supplementary file 1 — Supplementary file1 (DOCX 97 KB) [file 784_2023_5408_MOESM1_ESM.docx]

# **Clinical, Oral Immunological and Microbiological Shifts during and after Pregnancy**

Pınar Meriç, Angelika Silbereisen, Gülnur Emingil, Veli-Özgen Öztürk, Nagihan Bostanci


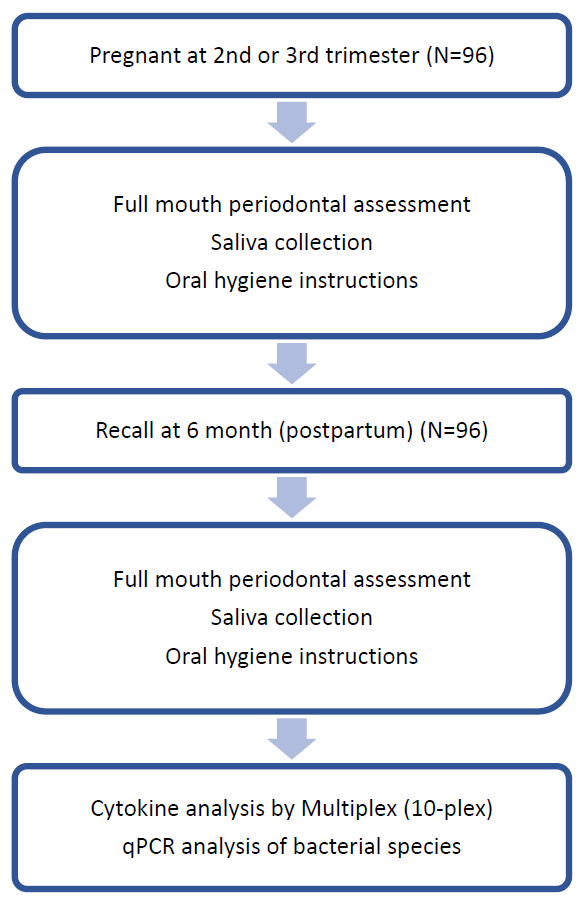
**SUPPLEMENTARY FILE**

**Supplementary Figure 1** Study outline. Sampling time and the follow up periods. Totally, 96 pregnant women were included in the study

**Supplementary Table 1** Clinical periodontal measurements in all study groups

|  | **Pregnant**  **N=96** | **Postpartum**  **N=96** |
| --- | --- | --- |
| **FMPD (mm)** | 2.0 (1.0) | 2.0 (1.0) |
| **FMBOP (%)** | 50.0 (42.5) | 50.0 (40.0) |
| **FMPI (%)** | 50.0 (50.0) | 50.0 (40.0) |
| **Baby Weight** | - | 3655 (636.25) |
|  | **Low birth weight**  **N=7** | **Normal birth weight**  **N=89** |
| **FMPD (mm)** | 1.0 (1.0) | 2.0 (1.0) |
| **FMBOP (%)** | 40.0 (50.5) | 50.0 (50.0) |
| **FMPI (%)** | 40.0 (50.0) | 50.0 (50.0) |

FMPD: Full mouth probing depth, FMPI: Full mouth plaque Index, FMBOP: Full mouth bleeding on probing. Median and IQR are given

**Supplementary Table 2** Biochemical data in the pregnant and postpartum groups

|  | **Pregnant**  **N=96** | **Postpartum**  **N=96** |
| --- | --- | --- |
| **Th1** | | |
| IL-2 (pg/ml) | 12 (15.92) | 15.6 (36.7) |
| INF-γ (pg/ml) | 1.1 (0.775) | 0.5 (1.9) |
| TNF-α (pg/ml) | 21.4 (23.8) | 20 (50.6) |
| **Th2** | | |
| IL-4 (pg/ml) | 72.1 (667) | 76.1 (107.9) |
| IL-5 (pg/ml) | 0.9 (0.7) | 1.3 (3.57) |
| IL-6 (pg/ml) | 4.75 (7.6) | 11.7 (42.27) |
| IL-10 (pg/ml) | 1.2 (1.1) | 1.85 (4.12) |
| **Proinflammatory cytokines** | | |
| IL-1β (pg/ml) | 177 (188) | 139 (137.5) |
| IL-8 (pg/ml) | 1788 (2611) | 1177 (1694) |

Median and IQR are given

**Supplementary Table 3** Biochemical and microbiological data in the low birth weight and normal birth weight groups

|  | **Low birth weight**  **N=7** | **Normal birth weight**  **N=89** | **p-value** |
| --- | --- | --- | --- |
| **Th1** | 31.3 (16.8) | 35 (41.6) | 0.243 |
| **Th2** | 63.4 (98.3) | 85.1 (83.3) | 0.717 |
| **Th1/Th2 ratio** | 0.406 (0.241) | 0.471 (0.255) | 0.081 |
| ***P. gingivalis*** | 197 (708) | 234 (4865.6) | 0.420 |
| ***T. denticola*** | 5998 (30546) | 3481 (16810) | 0.380 |
| ***T. forsythia*** | 47276 (717971) | 96245 (202442) | 0.989 |
| ***C. rectus*** | 62288 (397719) | 104449 (249360) | 0.912 |
| ***P. intermedia*** | 692 (2427) | 908 (11182) | 0.933 |
| ***F. nucleatum*** | 41846 (717726) | 148631 (325007) | 0.339 |

Th1: IFN-γ, IL-2, TNF-α, Th2: IL-4, IL-5, IL-6, IL-10. Median and IQR are given

**Supplementary Table 4** Th1, Th2, proinflammatory cytokines and Th1/Th2 ratio levels according to the periodontal status in pregnant and postpartum groups

| **Pregnant** | **Periodontally Healthy**  **N=16** | **Gingivitis**  **N=71** | **Periodontitis**  **N=9** | **p-value** |
| --- | --- | --- | --- | --- |
| **Th1 (pg/ml)** | 27.85 (32.33) | 31.60 (42) | 62.20 (92.55)* | **0.027** |
| **Th2 (pg/ml)** | 61.2 (83.32) | 83.5 (79.5) | 123.1 (73.05)* | **0.036** |
| **Pro-inflammatory cytokines (pg/ml)** | 1544 (2715.7) | 2162 (2729.5) | 3138 (4206) | 0.215 |
| **Th1/Th2 ratio** | 0.51 (0.24) | 0.43 (0.24) | 0.59 (0.44) | 0.089 |
| **Postpartum** | **Periodontally Healthy**  **N=16** | **Gingivitis**  **N=71** | **Periodontitis**  **N=9** | **p-value** |
| **Th1 (pg/ml)** | 23.15 (34.22) | 38.8 (114.4) | 40.9 (96.6) | 0.281 |
| **Th2 (pg/ml)** | 65.4 (102) | 108.1 (191.8) | 133.3 (262.4) | 0.177 |
| **Pro-inflammatory cytokines (pg/ml)** | 1317 (1691.8) | 1220 (1935.5) | 1333 (1846.4) | 0.946 |
| **Th1/Th2 ratio** | 0.38 (0.12) | 0.37 (0.12) | 0.36 (0.19) | 0.792 |

Th1: IFN-γ, IL-2, TNF-α, Th2: IL-4, IL-5, IL-6, IL-10, pro-inflammatory cytokines: IL-1β, IL-8. Median and IQR are given. *Significantly different from periodontally healthy and gingivitis groups (p<0.05)
